# Role of Tricellular Tight Junction Protein Lipolysis-Stimulated Lipoprotein Receptor (LSR) in Cancer Cells

**DOI:** 10.3390/ijms20143555

**Published:** 2019-07-20

**Authors:** Takayuki Kohno, Takumi Konno, Takashi Kojima

**Affiliations:** Department of Cell Science, Research Institute for Frontier Medicine, Sapporo Medical University, Sapporo 060-8556, Japan

**Keywords:** tricellular tight junctions, endometrial cancer, epithelial barrier dysfunction

## Abstract

Maintaining a robust epithelial barrier requires the accumulation of tight junction proteins, LSR/angulin-1 and tricellulin, at the tricellular contacts. Alterations in the localization of these proteins temporarily cause epithelial barrier dysfunction, which is closely associated with not only physiological differentiation but also cancer progression and metastasis. In normal human endometrial tissues, the endometrial cells undergo repeated proliferation and differentiation under physiological conditions. Recent observations have revealed that the localization and expression of LSR/angulin-1 and tricellulin are altered in a menstrual cycle-dependent manner. Moreover, it has been shown that endometrial cancer progression affects these alterations. This review highlights the differences in the localization and expression of tight junction proteins in normal endometrial cells and endometrial cancers and how they cause functional changes in cells.

## 1. Introduction

**Figure 1:**
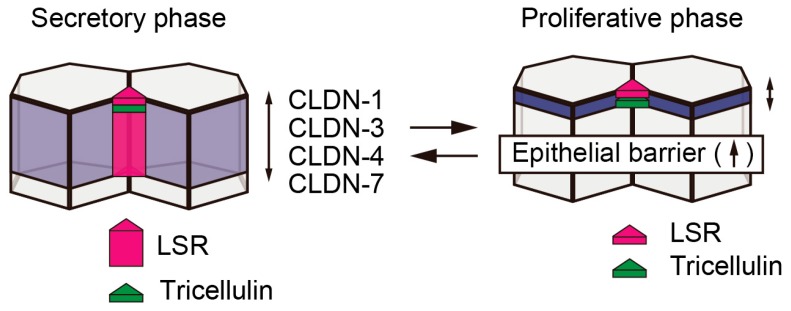
The localization of tight junction proteins is affected by menstrual cycle. In secretory phase of human endometrium, CLDN-1, -3, -4, and -7 are widely distributed to the lateral region. Tricellulin localized in tricellular contacts of the subapical region, whereas LSR is widely distributed to the lateral region. In proliferative phase, CLDNs localized in the subapical tight junction region. Tricellulin and LSR localized in the subapical tricellular contacts.

The endometrium is a regenerative tissue in which the cells undergo proliferation and differentiation depending on the levels of estrogen, progesterone, or various cytokines. The organization of cell-cell junctions, such as tight junctions, adherence junctions, gap junctions, and desmosomes, has important implications for the homeostatic regulation of many tissues, including the endometrium [1]. Cell-cell junctions are formed not only in bicellular regions but also at tricellular contacts [2]. Several reviews have mentioned that occludin (OCLN) and claudins (CLDNs) have been established as bicellular tight junction proteins involved in the formation and maintenance of epithelial barriers [3,4,5]. A recent study revealed that their expression and localization are affected by the menstrual cycle [6]. According to the report, CLDN-1, -3, -4, and -7 localized in the subapical region during the proliferative phase of the endometrium, while they were broadly distributed to the lateral region during the secretory phase (Figure 1). Furthermore, it has been shown that robust epithelial barrier formation requires localization of these tight junction proteins at the subapical region by analyzing primary cultured normal human endometrial cells. Recent studies have revealed that the localization of tricellular tight junction proteins, tricellulin and LSR/angulin-1, to tricellular contacts is required for epithelial barrier maturation based on the proper localization of OCLN and CLDNs [7]. A recent study demonstrated that tricellulin localized in the subapical region during the endometrial secretory phase, whereas LSR was broadly distributed to the lateral region [8]. In contrast, during the proliferative phase of endometrium formation, both proteins localized in the subapical region. Furthermore, analysis using primary cultured normal human endometrial cells revealed that localization of LSR to the tricellular contacts is required for the formation of mature epithelial polarity with sufficient barrier function. These findings suggested that LSR and tricellulin are closely related to the functional regulation of periodic morphological changes in the endometrial tissue. In the normal human endometrium, a part of the mechanism that regulates the localization and expression of tricellular tight junction proteins has been elucidated below.

## 2. Tricellular Tight Junction Proteins and Cancer

Many oncogenic processes are known to be involved in genetic instability based on failure of DNA mismatch repair pathways [9]. It is an established fact that the abnormal cell growth, dedifferentiation, and EMT are induced by the activation of oncogenes, such as Ras, and/or the inactivation of tumor suppressor genes, such as PTEN and p53 [10]. These adverse events, like a cancer metastasis, are certainly accompanied with reconstitution of cell-cell junctions [11]. While most of the differentiated epithelial cells have established tight junctions, disruption of tight junctions abolishes cell polarity and promotes dedifferentiation [3,12]. Furthermore, a decrease in epithelial barrier function is implicated in cancer cell invasion and metastasis [13]. Epithelial barrier homeostasis is disrupted by decreased expression of tight junction proteins as well as by their overexpression [14]. It still remains largely unknown how expression of tight junction proteins is regulated during the oncogenic process. Interestingly, decreased expression of tricellulin, which regulates epithelial barrier maturation, has been reported to be associated with tumor progression. For instance, in human tonsillar squamous cell carcinoma, decreased expression of tricellulin and CLDN-7 and increased expression of CLDN-1 have been identified [15]. In hepatocellular carcinoma cells, decreased expression of tricellulin has been observed as compared to that in normal hepatocytes [16]. In addition, lower prognosis of intrahepatic cholangiocarcinoma (iCCC) has been shown to correlate with decreased expression of tricellulin [17]. In pancreatic cancer, the decreased expression of tricellulin exhibits a correlation with decreased differentiation [18]. In gastric carcinoma, Snail-induced EMT negatively regulates the expression of tricellulin [19].

Increasing number of studies have reported the relationship between malignant transformation and expression of LSR, which is another tricellular tight junction protein. It has been reported that the expression of LSR is higher in invasive ductal carcinomas compared to that in invasive lobular carcinomas [20]. In addition, LSR is considered as a candidate prognostic biomarker in colon cancer patients [21]. Recent observations have revealed that the expression levels of LSR, tricellulin, and CLDN-1 were higher in head and neck squamous cell carcinoma tissues compared with those in normal palatine tonsils [22]. In addition, by analyzing the immunohistochemical staining using paraffin sections of head and neck squamous cell carcinoma tissue, it has been shown that the expression levels of both LSR and CLDN-1 are increased in cancerous tissues, especially in invasive tissues, compared to those in adjacent dysplasia tissues. Increased expression of CLDN-1 has been observed in advanced head and neck cancer [23]. CLDN-1 has also been shown to be significantly expressed in hypopharyngeal squamous cell carcinoma tissues, suggesting that CLDN-1 is associated with tumor differentiation and lymph node metastasis [24]. As described above, various cancerous malignancies are associated with changes in the expression and localization of not only bicellular tight junction proteins but also tricellular tight junction proteins. These findings suggested that tricellular tight junction proteins may interact closely with bicellular junctions during malignant transformation in response to reduction of the barrier function.

## 3. Expression and Localization of the Tricellular Tight Junction Proteins, LSR and Tricellulin, during Endometriosis and Endometrial Carcinoma

**Figure 2:**
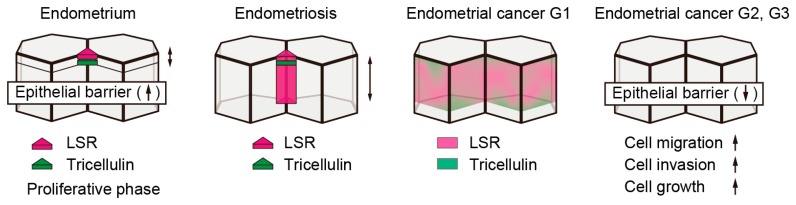
Expression and localization of LSR and tricellulin during endometriosis and in endometrial cancers. LSR and tricellulin localized in tricellular contacts in endometrium. During endometriosis, tricellulin is localized in the subapical region of tricellular contacts and LSR is localized in not only the subapical tricellular contacts but also in the lateral tricellular contacts. In endometrial cancer G1, tricellulin and LSR were distributed unevenly from the subapical to the lateral region of bicellular junctions. In endometrial cancer G2 and G3, the expression levels of tricellulin and LSR were downregulated, resulting in decrease of epithelial barrier and increase of cell migration, cell invasion, and cell growth.

During endometriosis, decreased expression levels of CLDN-3, -4, and -7 have been observed [25], and in endometrial cancer, increased expression levels of CLDN-3 and -4 have been reported [26,27]. Since changes in the expression levels of bicellular tight junction proteins were observed during the pathogenesis of the endometrial cancer, it is reasonable to consider that these processes were also accompanied by changes in expression levels of tricellular tight junction proteins. Recently, by analyzing the immunohistochemical staining using paraffin sections of endometriotic and endometrial cancer tissue, it has been found that during endometriosis tricellulin was localized in the subapical region similar to normal human endometrial tissue, while LSR was localized in the subapical region of tricellular contacts in addition to the lateral region [8]. In endometrial carcinoma G1, where the formation of gland-like structure is retained, the expression levels of tricellulin and LSR were distributed unevenly from the subapical to the lateral region of cell-cell junctions. In G2 and G3 endometrial carcinoma, their expression levels were decreased. Taken together, these findings revealed that the grade of malignancy correlated with the decreased expression levels of tricellulin and LSR in addition to changes in the localizations of these proteins (Figure 2). Among cultured cells derived from endometrial cancer, we were able to confirm the expression levels of both tricellulin and LSR in Sawano, HHUA, and JHMUE-1 cells, all of which exhibit an epithelial phenotype, whereas little or no expression was observed in JHMUE-2, which exhibits a fibroblast-like morphology. Since the expression levels of tricellulin and LSR contribute to the maintenance of the morphology of epithelial cells, we hypothesized that depletion of these proteins enhances cell motility. The endometrial cancer cell line, Sawano, endogenously expresses tricellulin and LSR. In Sawano cells with LSR knockdown, the epithelial barrier function was reduced, and thereby, cell motility, cell invasion, and proliferation were enhanced compared to those in the parental control. Thus, the localization of LSR at tricellular contacts is necessary for maintaining the robustness of the epithelial barrier function. The relationship between the exclusion of LSR from tricellular contacts and cancer progression has been discussed below, with a focus on endometrial cancer.

## 4. Obesity and Endometrial Cancer

Diagnoses of endometrial cancer have increased worldwide in recent years [28]. Obesity is a major risk factor for endometrial cancer [29]. Bioinformatics analysis using cBioProtal and DAVID bioinformatics resources confirmed that expression of genes related to glucose metabolism and lipid metabolism is increased in endometrial cancer [30]. Increase in estrogen, decrease in adiponectin, and increase in inflammatory cytokines are all known as typical cancer-inducing factors [31]. Leptin has also been reported to be involved in endometrial cell proliferation [32]. Previous studies have reported that an increase in circulating adiponectin and leptin-adiponectin ratio may be potential risk factors for breast cancer, colorectal cancer, pancreatic cancer, and endometrial cancer [33,34]. Leptin is produced not only from an adipose tissue, but also from follicles and placenta, and its production is associated with menstrual cycle and pregnancy [35,36]. Leptin is involved in facilitating endometrial cancer progression and metastasis of pancreatic cancer via the activation of JAK2/STAT3 pathway [37,38]. Adiponectin suppresses the progression and development of cancer by antagonizing this pathway [39]. It has been found that in endometrial cancer cells, leptin suppressed the expression of LSR, while adiponectin increased its expression [8]. Moreover, studies using inhibitors suggested that the stimulation with leptin or adiponectin induced an alteration of LSR expression via the PI3K and JAK2/3 pathways.

**Figure 3:**
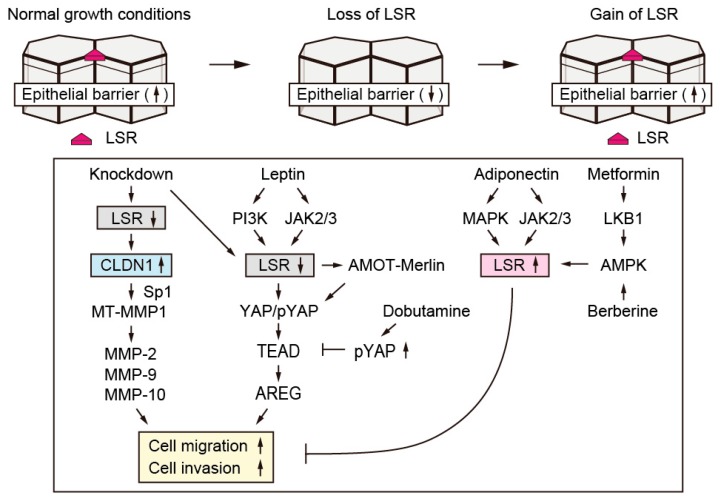
Changes in cellular functions by repression and re-expression of LSR. Under normal growth conditions, LSR localized in tricellular contacts in primary cultured normal human endometrial cells and Sawano cells. The knockdown of LSR enhanced cell motility and cell growth accompanying with decrease in barrier function. Leptin suppressed LSR expression; in contrast, adiponectin induced an increase in its expression. AMPK activator metformin and berberine also induced an increase in LSR expression at the subapical region of tricellular contacts, resulting in the rescue of the LSR-knockdown phenotypes.

It has been speculated that there is an interface between the regulatory pathways of the epithelial barrier formation and signaling pathways via the adipocytokine receptor. The knockdown of LSR enhanced cell motility and invasion in Sawano cells. This finding correlated with the cellular response associated with leptin-dependent downregulation of LSR (Figure 3). Interestingly, even in normal human endometrial cells, leptin suppressed LSR expression, while adiponectin increased its expression. It is assumed that obesity is involved in the malignant transformation of endometrial cancer besides attenuating the robust tight junctions of normal endometrium. LSR has been identified as a lipid receptor involved in lipid clearance [40]. In mice, suppression of LSR expression in the liver causes systemic hyperlipidemia, resulting in obesity and weight gain [41]. The differences in the function and role of LSR as a lipoprotein receptor and the involvement of LSR in obesity-dependent epithelial barrier attenuation should be clarified in future studies.

## 5. Glucose Metabolism and Endometrial Cancer

Obesity has been reported to be an independent risk factor for the development of diabetes [42]. Epidemiological studies have shown that metformin, a therapeutic agent for type 2 diabetes, reduces the incidence of endometrial cancer [43]. In addition, berberine, which is a herbal medicine component, has been reported to be not only effective in type 2 diabetes, but also in suppression of growth of cancer [44]. We found that metformin and berberine both increased LSR expression in endometrial cancer cells. The upregulation of LSR expression by these drugs contributed to the suppression of motility and invasion of endometrial cancer cells enhanced by leptin administration. Metformin and berberine also increased LSR expression in primary cultured normal human endometrial cells (Figure 3). Therefore, these drugs may be used to treat diseases based on epithelial barrier disruption. In fact, these drugs, which are categorized as AMPK activators, are currently being considered as potential therapeutic agents for endometrial cancer [43,44,45].

AMPK is an energy sensor that regulates the levels of intracellular ATP and centrally regulates metabolism [46,47]. Initially, depletion of intracellular ATP was reported to temporarily and reversibly disrupt tight junctions [48]. However, recent studies have indicated that AMPK, rather than affecting the intracellular ATP levels, may directly regulate tight junction proteins [49]. In the report, the authors revealed that AMPK regulates the relocalization of ZO-1 after Ca switch, independently of the intracellular ATP levels. Furthermore, AMPK has been reported to promote stabilization of tight junctions and to enhance barrier function via phosphorylation of the scaffold protein, GIV, which regulates cell polarity [50]. Metformin acts as a therapeutic agent for diabetes via LKB1-mediated phosphorylation of AMPK, which is accompanied by mitochondrial OxPhos suppression [51], suggesting that, in epithelial cells, metformin stabilizes tight junctions via the activation of AMPK. Interestingly, it has been previously reported that the progression of endometrial cancer correlates with the decrease in AMPK expression [52]. It is necessary to elucidate the signal transduction pathways involved in AMPK-regulated glucose metabolism and the regulation of epithelial barrier function.

## 6. Mechanisms of Enhancement of Cell Invasion Caused by Decreased LSR Expression

Using immunohistochemical analysis of paraffinized sections of endometrial cancer tissues, we observed a positive expression of LSR and negative expression of CLDN-1 in the gland-like structure region. In contrast, in the invasive front area, LSR expression decreased and CLDN-1 expression increased. Following knockdown of LSR in endometrial cancer Sawano cells, CLDN-1 expression increased, while there was no significant change in the expression levels of CLDN-3, -4, -7, and OCLN. Before LSR knockdown, although CLDN-1 localized in the subapical region, it was widely distributed not only to the subapical region but also to the lateral region after LSR knockdown. These findings suggested that there was a negative relationship between the expression levels of LSR and CLDN-1 (Figure 3). In intestinal epithelial cells, it has been reported that regulation of CLDN-1 expression requires Sp1 binding to the CLDN-1 promoter region [53]. It has been reported that CLDN-1, -4, and -19 harbor Sp1 binding sites in the promoter region [54,55,56]. We confirmed that Sp1-dependent transcriptional regulation was involved in the enhancement of CLDN-1 expression associated with LSR repression [57]. 

It has been reported that cell invasion is enhanced via the cleavage of laminin-5 gamma 2 chains by activation of MT-MMP1 and MMP2 in CLDN-1-overexpressing OSC cells [58]. In addition, in SW480 cells, overexpressing CLDN-1, cell invasion is enhanced through the activation of MMP2 and MMP9 [59]. The initial process of cell invasion requires reconstitution of extracellular matrix components, along with the attenuation of cell junctions [60]. Twenty four MMP family members have been identified so far [61]. It has been found that knockdown of LSR increased the expression levels of MT-MMP1, MMP2, MMP9, and MMP10 in Sawano cells [57]. MT-MMP1 has been reported to be a initiating factor that regulates the MMP cascade following the activation of proMMP2 [62]. Interestingly, double knockdown of LSR and CLDN-1 suppressed the increase in cell invasion by LSR knockdown [57]. Little is known about the precise molecular mechanisms underlying the activation of MMPs accompanying the expression of CLDN-1 in endometrial cancer tissues. The suppression of LSR downregulation may regulate the malignant transformation of endometrial cancer.

## 7. Hippo Pathway and Endometrial Cancer

Relaxation of cell-cell junctions and abnormality of epithelial polarity suppress contact inhibition in epithelial cells, resulting in the induction of abnormal proliferation. The Hippo pathway comprehensively regulates these mechanisms [63]. When the Hippo pathway is turned on, LATS1/2 is phosphorylated via MST1/2. Phosphorylated LATS1/2 phosphorylates YAP, and phosphorylated YAP is degraded via 14-3-3. On the other hand, when the Hippo pathway is blocked, the phosphorylation of YAP is suppressed. The non-phosphorylated form of YAP translocates from the cytoplasm to the nucleus as a transcription cofactor and induces the expression of target genes, such as AREG and DKK1, depending on the expression of the transcription factor TEAD.

We found that YAP is localized in the cytoplasm of the endometrial tissue and in the nucleus in G1, G2, and G3 endometrioid carcinoma, as revealed by immunohistochemical staining using paraffinized sections of endometriotic and endometrial cancer tissues. As mentioned above, cell motility and invasion enhanced by knockdown of LSR were decreased by double knockdown of LSR and YAP. These findings suggested that the decrease in epithelial barrier function caused by the suppression of LSR expression is involved in the regulation of cell motility and invasion via YAP (Figure 3). The β-adrenergic receptor agonist, dobutamine, decreases nuclear YAP levels and increases the amount of cytosolic phosphorylated YAP in human osteoblastoma U2OS cells [64]. Dobutamine has also been reported to suppress the enhancement in the expression of YAP in gastric carcinoma, resulting in the suppression of cell motility and invasion [65]. In addition, in LSR-knocked down Sawano cells, dobutamine administration suppressed the enhancement in cell motility and invasion via the increase of phosphorylated YAP. The precise molecular mechanisms underlying the phosphorylation of Hippo kinases, such as MST1/2 and LATS1/2, via LSR-mediated epithelial barrier modulation still need to be elucidated.

Under glucose starvation conditions, AMPK is phosphorylated by LKB1 [66]. Phosphorylated AMPK has been reported to suppress nuclear translocation of YAP via the phosphorylation of LATS1/2 and/or direct phosphorylation of YAP [47]. In Sawano cells, under glucose-starving conditions, YAP is localized in the proximity of the cell-cell junctions [67]. In addition, both AMPK and YAP were phosphorylated. Moreover, both cell invasion and cell motility enhanced by LSR knockdown were rescued by glucose starvation. It has been speculated that these mechanisms are probably similar to the effect of treatment of AMPK activator, metformin or berberine, as mentioned above. Glucose starvation also increased LSR expression. Further studies are needed to elucidate the signaling pathways by which glucose starvation regulates epithelial barrier functions in endometrial cancer.

Using DNA microarray and qPCR analysis, it has been found that the expression levels of the transcription factors TEAD and AREG, increased in LSR-knockdown Sawano cells [67]. Moreover, immunohistochemical analysis using paraffinized sections of endometriotic and endometrial cancer tissue showed that AREG was expressed in the cytoplasm and that the expression increased with the progression of cancer stage. In Sawano cells, increasing cell motility and invasion by LSR knockdown was suppressed by knockdown of AREG. These effects were also observed after TEAD knockdown. In parental Sawano cells, knockdown of AREG did not affect cell motility and invasion. Therefore, it is concluded that TEAD-dependent AREG expression via the Hippo pathway is involved in the enhancement of cell motility accompanied by the suppression of LSR expression.

## 8. Crosstalk between the Hippo Pathway and Tight Junctions

Merlin/NF2 is known as one of the tumor suppressor factors that regulate the Hippo pathway [68]. Merlin localizes to adherens junctions by interacting with E-cadherin, PAR3, and catenin [69]. Merlin also interacts with YAP and AMOT, a scaffold protein of Mst1/2 and LATS1/2 at tight junctions and contributes to the regulation of EMT [70]. It has been suggested that changes in the cell adhesion between adjacent cells, that is, modulation of tight junctions and adherens junctions, regulate the phosphorylation of YAP via the Hippo pathway, leading to the disruption of contact inhibition and normal growth. However, the precise molecular mechanisms have yet to be elucidated.

By immunohistochemical analysis, we found that AMOT localized in the subapical region and the lateral region of endometriosis tissues [67]. In endometrioid adenocarcinoma, positive expression of AMOT was observed in the gland-like structure region. Compared with that in endometrial carcinoma G1, decreased expression of AMOT was observed in G2 and G3. The Motin family consists of AMOT (angiomotin), AMOTL1 (angiomotin-like 1), and AMOTL2 (angiomotin-like 2) [71]. In addition, two isoforms of AMOT, AMOT-p130 and AMOT-p80, have been identified. AMOT-p80 has been identified as an oncogene in hemangioendothelioma, head and neck squamous cell carcinoma, and prostate cancer [72,73,74]. AMOT-p130 has been reported to exhibit oncogenic functions as well as tumor suppressive functions [71]. AMOTL1 has been shown to act as an oncogene in breast cancer [75] and cervical cancer [76], and AMOTL2 has been reported to act as an oncogene in breast cancer [77] and suppressor glioblastoma carcinogenesis [78]. In endometrial cancer, decreased expression of AMOT was observed during cancer progression [67]. Molecular mechanisms related to AMOT in endometrial cancer would be clarified in the near future.

Using immunostaining analysis, we found that, in Sawano cells, endogenous Merlin localized in the vicinity of the cell-cell junctions identically to the other cells [68,79]. Under these conditions, AMOT is localized in tight junctions. It is known that AMOT interacts with Patj, Pals2, and Mupp1 at the tight junctions and that Merlin binds to the coiled-coil region of AMOT [80]. The Rac GTPase-activating protein, Rich1, binds through this region of AMOT. In mature tight junctions because Merlin binds to AMOT, Rich1 cannot interact with AMOT and localizes to the cytosol, resulting in the inactivation of Rac. When Merlin is dissociated from AMOT, which is localized at tight junctions, Rich1 binds to the coiled-coil region of AMOT, thereby activating Rac and enhancing cell proliferation and cell motility. In Sawano cells, LSR knockdown decreased the expression levels of both AMOT and Merlin, and AMOT and LSR double knockdown further reduced the expression of Merlin [67]. The report revealed that the increased invasion and motility of Sawano cells by LSR knockdown was suppressed by AMOT knockdown. In the parental Sawano cells, AMOT knockdown increased cell invasion and motility, which were, in turn, suppressed by YAP knockdown. These findings suggested that YAP as well as Rac are involved in the malignant transformation of endometrial cancer cells. Identification of the crosstalk between AMOT/Merlin pathway and Rac/Rich1 pathway is considered to contribute to the elucidation of the malignant transformation mechanisms of endometrial cancer.

## 9. Changes in LSR Localization Are Associated with Changes in Cell Size Following Changes in Cell Density

An inverse correlation has been reported between cell density and motility of epithelial cells [81,82]. At high cell densities, apparent cell size decreases with increase in cell thickness accompanying decreasing cell motility. Conversely, at low cell densities, cells spread thinly and cell motility increases. In Sawano cells, LSR and tricellulin localized in tricellular contacts under high cell density conditions, while these proteins migrated to the bicellular region under low cell density conditions despite the presence of tricellular contacts [83]. Under these conditions, TER measurements indicated that an epithelial barrier was present even at low cell densities. The localizations of the bicellular tight junction proteins, OCLN and CLDN-4, were not affected by changes in cell density. Furthermore, knockdown of LSR under high cell density conditions induced thin spreading of cells and enhanced cell motility (unpublished observation). These findings suggested that the localization of LSR at tricellular contacts is necessary for the maintenance of static epithelial cell sheets.

**Figure 4:**
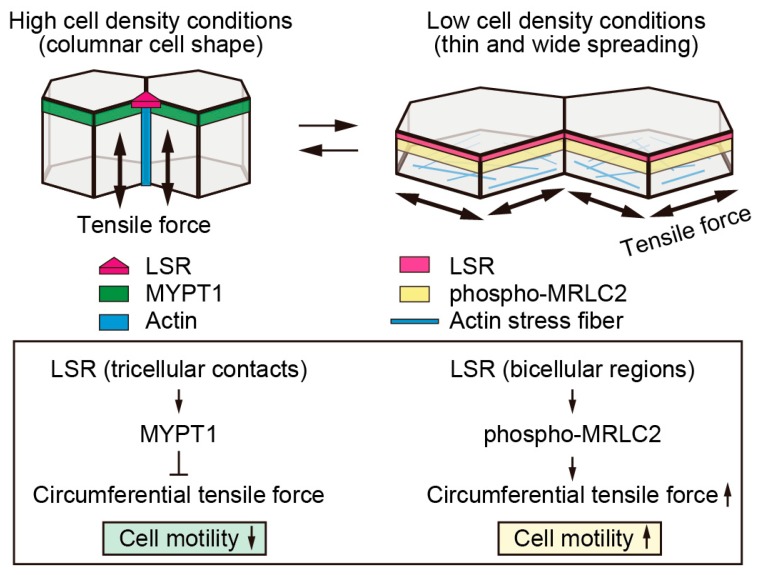
Decreased cellular tension causes LSR to localize at tricellular contacts. At high cell density, where cells were grown with columnar shape, the cellular tension decreased in the circumferential direction because MYPT1 is localized in these regions. Under these conditions, LSR is localized in tricellular contacts. In contrast, higher tension existed in cell-cell junctions at lower cell density because the phosphorylated form of MRLC2 is localized in these regions. In addition, actin stress fibers formed adjacent to the basal membrane. Under these conditions, LSR localized in bicellular junctions. It is noteworthy that thin and wide spreading cells increase cell motility.

An increase in cell density affects the intracellular tension mediated by actomyosin [84]. MRLC2 is localized at bicellular regions in the low density culture of Sawano cells [83]. Phosphorylated form of MRLC2, which represents activated myosin, is also localized in these regions. Under high cell density conditions, MRLC2 accumulated in vesicles or aggregated as particles near the apical cell surface membrane without localization to the apical bicellular region. It has been reported that activated MRLC2 is dephosphorylated by MYPT1, which is a component of the myosin phosphatase complex [85]. MYPT1 is localized in bicellular regions under high cell density conditions; however, under low cell density conditions, this protein was largely delocalized. These findings suggested that there is a high intracellular tension at bicellular regions of low density-grown cells as compared to that of high density-grown cells. When phosphorylated MRLC2 is localized in bicellular regions and MYPT1 is delocalized from there, LSR is localized in bicellular tight junctions. On the other hand, when MYPT1 is localized in bicellular regions, LSR is localized in apical tricellular contacts (Figure 4). Taken together, the localization of LSR altered in a cell density- and/or cellular tension-dependent manner. It is necessary to elucidate how LSR recognizes cell size and intracellular tension.

## 10. Decreased Cellular Tension Causes LSR to Localize at Tricellular Contacts

Cellular tension is formed at cell-cell junctions and cell-substrate interface [86]. When the cells occupy a wide spread area, the number of focal contacts, where paxillin and integrins bind to the extracellular matrix, increases at basal membrane, facilitating polymerization of actin cytoskeleton [87]. Myosin, a cross-linked protein of actin fibers, generates cellular tension by contracting actin fibers [88]. Sawano cells cultured at low density conditions were spread thin and wide, and significant stress fiber formation was induced [83]. This suggested that high cellular tension is present under these conditions. Cellular tension is reportedly reduced by ROCK inhibitor, Y27632, muscle and non-muscle myosin II inhibitor, blebbistatin, or MLCK inhibitor, ML-7 [89]. When these reagents were added under low density conditions of Sawano cells where LSR localized in bicellular junctions, LSR localization decreased at bicellular junctions and increased at tricellular contacts. In our preliminary experiments, the focal adhesion kinase FAK localized in bicellular regions under low density of Sawano cells. FAK has been reported to be involved in the control of intestinal barrier functions [90] and blood-testis barrier functions [91]. In Sawano cells, FAK was dislocated from bicellular regions with increasing cell densities; however, the underlying mechanisms are still unclear. It is thought that the relationship between the regulatory mechanism of cellular tension involving FAK and the regulatory mechanism of epithelial barrier functions involving LSR will become clear in future studies.

## 11. Crosstalk between Intracellular Tension and Cell Junctions

In Sawano cells, LSR is reversibly translocated in a cellular tension- and/or cell size-dependent manner, whereas the localizations of OCLN and CLDN-4 were not affected by these conditions. Interestingly, accumulation of F-actin was observed not only along the lateral region of tricellular contacts but also the circumferential subapical region, leading to increase in cell thickness with the high cell density culture of Sawano cells [83]. Moreover, the actin polymerization inhibitor, Cytochalasin D, excluded LSR from tricellular contacts, resulting in partial stratification of monolayered Sawano cell sheets. In long-term high-density culture, Sawano cells spontaneously and partially stratified in absence of Cytochalasin D. Under these conditions, the accumulated F-actin disappeared from lateral regions of tricellular contacts near the stratified area. In addition, LSR was translocated from tricellular contacts to the cell surface besides intracellular vesicles. Simultaneously, the epithelial barrier function decreased (unpublished observations). These findings suggested that the accumulation of F-actin at tricellular contacts is implicated in LSR localization to tricellular contacts. It is still not clear how the accumulated actin at tricellular regions regulates cellular tension. It is necessary to analyze the precise mechanism by which LSR localization is regulated by tension formed by actomyosin at tricellular contacts. 

There have been almost no reports that LSR interacts directly with the actomyosin cytoskeleton at tricellular contacts. In contrast, it has been revealed that tricellulin promotes localization of both actin and myosin at tricellular contacts via interaction with Cdc42GEF protein Tuba (DNMBP, ARHGEF36) [92]. Cdc42 is one of the key proteins involved in the formation and maturation of epithelial polarity and contributes to the enhancement of cellular tension via MRCK(Cdc42BPA) [93]. MYPT1 is inactivated via phosphorylation by MRCK or ROCK [94,95]. In addition, the accumulation of tricellulin at tricellular contacts is controlled by LSR [7,96]. However, little is known about the downregulation of cellular tension during epithelial cell maturation by these proteins. Reportedly, the non-phosphorylated form of LSR in which mutations have been introduced to serine residues localizes to bicellular junctions [97]. However, the significance of LSR localization at bicellular junctions and its underlying molecular mechanism have not been elucidated yet. It is thought that changes in LSR localization along with changes in cellular tension are associated with acquisition of endometrial cancer motility. Further studies are required in order to understand the precise molecular mechanisms underlying regulation of LSR localization based on the changes in actomyosin activity and cellular tension.

Adherens junctions organize prior to tight junctions during the intercellular closure [98]. Adherens junctions interact with the actin cytoskeleton via the nectin-afadin complex and the cadherin-α/β-catenin complex [4]. Thereby, the actin cytoskeleton forms circumferential actomyosin bundles, contributing to cell polarity and cell thickness [99]. Shroom3 and Lulu1/2 are involved in the regulation of circumferential actomyosin bundles via ROCK [100,101]. Tight junctions are organized by the polarity complex consisting of PAR3, PAR6, and aPKC, which interact with circumferential actomyosin bundles via ZO1/2/3 [102]. For polarized epithelial cells, the cell thickness increases with long-term culture. The process of cell thickening is involved in the rearrangement of the actin cytoskeleton and the microtubule network [103]. Drebrin has been identified as a factor linking the actin cytoskeleton to the microtubule network through the interaction with complex consisting of myosin IIB, spectrin βII, and EB3 [104]. The report has revealed that, in Drebrin knockout cells, the cell thickness was reduced without compromising the cell polarity and the barrier function. Drebrin also localizes to gap junctions by interacting with connexin-43 [105]. Further studies are required to investigate whether Drebrin regulates LSR localization.

## 12. Hippo Pathway and Cytoskeletal Dynamics

Junctional complexes comprised of tight junctions and adherens junctions are important for the maintenance of apico-basal polarity in planar epithelial cells [4]. Many of these complexes interact with various adaptor proteins via PDZ domains and are linked to actomyosin networks [106]. These interactions allow epithelial cells to maintain apico-basal polarity [107]. Insufficient planar cell polarity is formed when only a few cells exist in a population per unit area [4]. In contrast, planar cell mobility decreases as the number of cells increases in the population until contact inhibition occurs, thereby resulting in apico-basal polarity formation [108]. It is already known that the Hippo pathway participates in these processes. In polarized epithelial cells, the Hippo pathway is involved with the maturation of tight junctions and adherens junctions, as well as the planar cell polarity pathway, mechanotransduction pathway, and growth factor signaling [82]. YAP (yes-associated protein) is a regulator of cell size [109]. This report revealed that a signal from widely spread cells induced the activation of transcription factors following the nuclear localization of YAP, whereas a signal from narrowly spread cells induced the inhibition of transcription factors following the degradation of YAP outside the nucleus [110]. Moreover, ARHGAP18, a Rho GTPase-activating protein, regulates the cortical actin network through the YAP signaling cascade [111]. A recent study has indicated that in endometrial carcinoma, the nuclear localization of YAP is involved in increased malignancy [112]. MYPT1 is known to activate Merlin [68,79]. The localization of MYPT1 was sensitive to changes in cell density [83]. Therefore, in endometrial cancer cells, the activity of Merlin might be altered by changes in cell density. It is necessary to identify the molecules involved in the crosstalk between the mechanism that regulates the cell size based on YAP expression and the mechanism that regulates the tight junction integrity based on the cellular tension.

## 13. Conclusions

During endometrial cancer progression, a decreased expression of LSR and increased expression of CLDN-1 have been observed. In primary cultured normal human endometrial cells, leptin reduced the expression of LSR. Obesity is one of the risk factors for endometrial cancer. These findings demonstrated that disruption of epithelial barrier integrity due to translocation of LSR was related to the mechanism of malignant transformation of endometrial cancer. These mechanisms were closely related to the Hippo pathway and also involved the reconstitution of extracellular matrix components. Furthermore, changes in cellular tension were associated with changes in LSR localization. The Hippo pathway has also been reported to be involved in the regulation of cellular tension. The cellular tension influences the translocation of LSR from bicellular junctions to tricellular contacts. On the contrary, the mechanism that causes the disruption of the robust epithelial barrier is poorly understood in endometrial cancer cells. In order to elucidate functional changes in the epithelial barrier during the malignant transformation of normal endometrial cells, it is required to accurately analyze the molecular mechanisms that regulate the localization of tricellular tight junction proteins.

## Abbreviations

CLDNs	claudins
EMT	epithelial-mesenchymal transition
HCC	hepatocellular carcinoma
HNSCC	head and neck squamous cell carcinoma
iCCC	intrahepatic cholangiocarcinoma
LSR	lipolysis-stimulated lipoprotein receptor
MLCK	myosin light chain kinase
MYPT1	myosin phosphatase target subunit 1
MRLC2	myosin regulatory light chain 2
OCLN	occludin
OSC	oral squamous cell carcinoma
TER	transepithelial electrical resistance
